# Vital Chemistry—Lectures on Animal Heat

**Published:** 1845-08

**Authors:** 


### ILLINOIS
### MEDICAL & SURGICAL JOURNAL.

VOL. II. AUGUST, 1845. NO. 5.

(REVIEW.)

*Vital Chemistry—Lectures on Animal Heat.* By THOMAS SPENCER, M. D., Prof. of the Institutes and Practice of Medicine, in the Medical Institution of Geneva College. Published by request of the Class. Geneva, 1845. pp. 114.

The above work does not assume to present any new experimental facts upon the subject, now much agitated, of Animal Heat, but a discussion of the subject, founded upon facts, for which the author acknowledges himself indebted "to those who have more particularly devoted their attention to these subjects, while the deductions are the result of his own reflections."

The new impulse given to the discussion of this most interesting subject by the investigations of Liebig, and the theories founded thereon, has called into the field two classes of disputants—the votaries of the chemical theories, and the advocates of, so called, pure physiology. As usual, both parties have carried their views beyond the limits of experimental truth and legitimate induction. The former class gives to chemical action an undue importance in the animal economy, allowing too little influence to vital force, in the modification of the laws applicable only to the mineral kingdom. The latter class, by denying entirely the agency of chemical force, as adjuvant to vital power, discourage investigation, and check the advance of science.

We are inclined to think that by both these classes, certain forces which perhaps may be called mechanical, have received less attention than they deserve. As an instance of that to which we refer, may be cited the force which causes the absorption of oxygen and the escape of carbonic acid in respiration. This appears to us to be due simply to the diffusive force of gases, as established by Prof. Graham.

Dr. Spencer, in the lectures before us, assumes the presence of a triple compound of carbon, hydrogen, and oxygen, in venous blood. This compound he considers analagous to lignin or humus, and causes it to combine, during respiration, with oxygen; the carbon forming carbonic acid, and the hydrogen and oxygen escaping as vapor of water. The author appears sensible of the possibility of the heat thus generated consuming the lungs, and attempts to dispose of the large excess by the absorption of heat, in the exhalation of pulmonary vapor. That this is totally inadequate to the task assigned it, becomes evident, when we consider that in ordinary combustion of wood, its combined water, when passing into the state of vapor, absorbs an *imperceptible* amount of the sensible heat. It is only the water combined in the "hydrate of carbon," to which the author assigns the duty of preventing combustion of the lungs. If this be the only means of attaining the end required, the objection must still stand, that the combustion of the amount of carbon passing off by the lungs in the carbonic acid expired, if taking place in these organs, would elevate their temperature far beyond their natural state, if not to such a degree as to destroy the tissues. Besides, the mass of evidence in favor of the existence of carbonic acid, *as such*, in venous blood, and in proportion much greater than in arterial, appears to us conclusive, notwithstanding the Professor's answer to the objection in his last chapter. If this be true, there is no necessity for supposing, with Dr. Spencer, that combustion of carbon actually takes place in the lungs. Another proof of the same, is the fact that carbonic acid is equally expired, if gases *containing no oxygen* be inspired, as shown by the experiments of Spallanzani on cold-blooded animals, since repeated, with the same results, by M. Edwards. (See Müller's Physiology, book III, chap. 5.) This objection is ingeniously answered by Dr. S., in his last chapter, but the following, which is still more opposed to his theory, and we think unanswerable, he does not notice. The simple agitation of venous blood, out of the body, with hydrogen, nitrogen, and other gases *contain-*

*ing no oxygen*, is followed by the same result. How, we ask, can this be accounted for, otherwise than by the fact that the mechanical force of diffusion liberates the carbonic acid *existing as such?* Recent experiments of Mulder, as reported by Dr. Golding Bird, (see New York Jour. of Med., vol. IV, p. 409,) refer, with much reason, and in consistency with facts established by Scherer and Hewson, the change from the venous to the arterial hue, to the mechanical effect of various circumstances in modifying the reflective and refractive power of the fluids, with regard to light. In the work before us, no reference is to be found to Mulder's researches, which we think fatal to a large portion of the theory.

As regards the existence, assumed by Dr. Spencer, of the triple compound of carbon, hydrogen, and oxygen, in venous blood, we have only to say, that this is not proven by analysis, and as regards the coloring matter, it is distinctly proven, that it contains no more carbon in venous than in arterial blood.

The portion of the work discussing the agency of the compounds of iron in the function of hœmatosis, must yield, if we give any credit to the recent investigations of Mulder, (loc. cit.,) proving that iron exists in hœmatosine in the *metallic state*. This he proves by the fact, not before hinted at, that upon the digestion of blood globules with sulphuric acid, hydrogen escapes, which could not occur, if the iron existed combined. And by the same analysis it is shown, that the iron is *unessential to the red color of blood corpuscules.* It is but justice to our author to add, that to the best of our knowledge, the investigations last referred to, had not been published in this country, at the time of the issue of his work. As our object, however, and that of our author, is to find the truth, we have thought proper to present these, as worthy of consideration, in connection with his views.

We may notice in passing, a misunderstanding of a chemical principle quoted from Liebig, and to be found on page 58. The principle is, that "carbonate of protoxide of iron, in contact with water and oxygen, is decomposed; all the carbonic acid is given off, and by absorption of oxygen it passes into the hydrated peroxide." In making use of this fact, the author remarks: "The blood always contains *water*. The carbonic acid itself contains *oxygen*, and these two conditions of the law quoted (the presence of water and oxygen) existing, the carbonate of the protoxide of iron would be resolved into the hydrated peroxide, and the carbonic acid set free to combine in a new form." Every chemist will perceive that the author has entirely mistaken the law, it being absolutely essential to the reaction, that the oxygen itself be *free from all combination.* That carbonic acid will yield its own oxygen, to decompose one of its own compounds, is a chemical absurdity. Yet the establishment of the reaction quoted, is a most important link in the author's "circle of vital affinities," which indeed cannot be established without it. The author, on page 64, causes the reverse operation to occur in the lungs, when the conditions of the same law are actually present, *free* oxygen being supplied by inspiration.

Chapters VII and VIII discuss the chemico-vital relations of the pulmonic and gastric functions, and the chemico-vital connection of Digestion, Hepatic Secretion, Calorification, and Nutrition. They exhibit evidence of much reading and reflection, as well as ingenuity in connecting the various links of the chain of reactions. They are we think, subject to some objections, which we will state as briefly as possible. Chloride of soda, according to the author, is decomposed in the capillaries of the stomach, by a process analagous to galvanic action. The hydrochloric acid is secreted for the gastric juice, while the soda passes, one portion into the blood, to give it its alkaline reaction, another portion to the liver, to be used in the secretion of bile. A part of the hydrochloric acid is used to form sesquichloride of iron, which is supposed to pass by the lacteals into the blood, and on to the lungs, where it is decomposed by the free soda of the blood, forming common salt, and hydrated sesquioxide of iron. The author does not inform us why the chemical reaction, between the sesquichloride and the soda, does not take place in the subclavian vein, where the chyle reaches the blood, and what peculiar force compels it to wait for its reaction until it reaches the pulmonic capillaries. Again, the soda which passes to the liver, goes with the bile to the duodenum, there it finds albumen, which it dissolves, and passing into the lacteals, is carried into the blood. How, we would ask, can it pass through the lacteals, which are also made to carry the sesquichloride of iron, without the reaction taking place which would form common salt and sesquioxide of iron. In the next place, the hydrochloric acid of the gastric fluid, is made the solvent of fibrine and caseine, which solution also passes into the lacteals, through the usual route to the mass of the blood, and so on to the "systemic capillaries," where it meets with soda holding albumen in solution, common salt is produced, and the albumen, fibrine, and caseine are dropped for the purposes of nutrition. This is an exceedingly ingenious solution of the problem of nutrition, but we cannot see, and are not informed by the author, by what force the usual chemical reaction, between the hydrochloric acid and the soda, is arrested until it reaches the systemic capillaries. Why does it not occur in the intestinal canal, when the soda of the bile, and the acid of the chyme are brought together? 2d. Why does it not occur when they commingle in the lacteals? And 3d, Why not in the mass of the blood? The presumption of the author is, evidently, that this chemico-vital change can only occur in the two systems of capillaries. Yet he does not give adequate reasons for the hypothesis, merely implying it by his results.

In our remarks we have endeavored to do justice, both to the author, and to the cause of truth and science. We were not only willing, but desirous, to find in Dr. Spencer's work, a new and satisfactory solution of the problem he professes to elucidate; and regret that to notice the work, compels us to notice some discrepancy with established facts, and true chemical principles. This is due to the fact, that *experimental* chemistry has not, as Dr. S. acknowledges, been to a great extent the subject of his studies. In chemical research, speculation is not admissible. Careful experiment is requisite at every step. To assume one point, not confirmed by the most strict analytical proofs, is sufficient to admit doubts, if not to justify the rejection of the whole. Much arduous labor in reading and reflection, will, we fear, be lost upon the work under review, for the want of experimental confirmation. Much credit is due to the author for the originality of some of his views, and the industry and ability evinced in amassing the facts, and conducting the reasoning to its conclusion. It is only the failure to establish certain steps, and occasional misapprehension and oversight, that set aside the results.

We hope the investigation will be continued; for if failing ultimately in establishing the desired solution, it cannot fail to elucidate the subject to a greater or less extent, and incite experimental inquiry in others.—Ed.

